# Phenological variations of avocado cv. Hass and their relationship with thermal time under tropical conditions

**DOI:** 10.1016/j.heliyon.2023.e19642

**Published:** 2023-09-03

**Authors:** Joaquín Guillermo Ramírez-Gil, Juan Camilo Henao-Rojas, Cipriano Arturo Diaz-Diez, Andrés Javier Peña-Quiñones, Nicolas León, Alfonso Parra-Coronado, Jorge Alonso Bernal-Estrada

**Affiliations:** a Universidad Nacional de Colombia, Sede Bogotá, Facultad de Ciencias Agrarias, departamento de Agronomía, Bogotá 111321, Colombia; b Laboratorio de Agrocomputación y Análisis Epidemiológico, Center of Excellence in Scientific Computing, Universidad Nacional de Colombia, Bogotá 111321, Colombia; c Corporación Colombiana de Investigación Agropecuaria, AGROSAVIA, Centro de Investigación La Selva, Colombia; d Corporación Colombiana de Investigación Agropecuaria, AGROSAVIA, Centro de Investigación La Libertad, Colombia

**Keywords:** Base temperature, Altitudinal profile, Growing degree days, Phenological variability

## Abstract

In recent years, the avocado has been one of the most dynamic fruits in the world market. In particular, cv. Hass stands out due to its productivity, nutritional quality, and acceptance. Under tropical conditions, weather elements, especially air temperature, and precipitation, affect the productivity and quality of cv. Hass. However, in tropical environments, many relationships between weather and phenological aspects of this cultivar are still unknown. Given this situation, our aim was to identify the variation and degree of association between the phenology of avocado cv. Hass and thermal time (TT) under low-latitude conditions. Eight commercial fields planted with cv. Hass grafted onto Antillean genotypes, located in an altitudinal transect between 1,700 and 2,500 m, were evaluated. The evaluation was carried out for three years and was focused on determining the differences in avocado phenological patterns associated with different environmental variables monitored by weather stations at each location. Air temperature data were used to calculate the base temperature (BT) using different methods for all phenological stages. Later the TT was determined for each stage and all locations. The results show that the duration of each phenological stage varies as a function of elevation (air temperature) and that the phenological stages overlap at the regional, crop field, and plant levels at different periods of the year, generating a high phenological variability but with specific patterns associated with temperature and precipitation. The BT for each phenological stage varied between 0.3 and 7.5 °C, and TT was found to vary depending on the method of calculation. Our work suggests that the generalization of a BT of 10 °C is not applicable for avocado cv. Hass crops under tropical conditions, specifically in the case of Colombia. Detailed studies of phenological relationships with respect to climatic variables will allow a better approximation of the productive behavior of avocado cv. Hass.

## Highlights

• Hass avocado is subject to high variation in phenological stages under tropical conditions.
• We demonstrated that the generalization of a base temperature of 10 °C does not apply under tropical conditions, specifically in the case of Colombia.
• The duration of each phenological stage varies as a function of elevation (air temperature) and precipitation under tropical conditions.
• Phenological stages overlap at the regional, crop field, and plant levels, generating a high phenological variability.

## 1 Introduction

Avocado (*Persea americana* Mill.) is a species belonging to the Lauraceae family, which includes three currently described botanical races: Mexican (*P. americana* var. drymifolia), Guatemalan (*P. americana* var. guatemalensis Williams) and West Indian (*P. americana* var. americana Mill.) [1]. Multiple cultivars have originated from these races, thus increasing their range of distribution and adaptation. This has allowed commercial varieties to be widely cultivated in tropical, semitropical, and subtropical climates [2,3]. The cv. Hass stands out as one of the most widely cultivated and distributed varieties worldwide, due to its multiple advantages, such as productivity, nutritional quality, postharvest life, market acceptance, and the possibility to produce this in diverse environmental conditions [2,4]. The cv. Hass is planted mainly in three climatic zones: (i) cool, semi-arid climates with predominant rainfall in winter, such as California, Peru, Chile, and Israel; (ii) humid subtropical climates with predominant rainfall in summer, such as in Mexico, South Africa, and eastern Australia; and (iii) tropical and semi-tropical climates with rainy summers, such as in Brazil, Florida, Indonesia, Colombia, among others [3].

The cultivation of avocado cv. Hass has gained importance in recent years due to the increase in world demand [5]. Several factors have boosted the consumption of this fruit, mainly its high nutritional value [6] and beneficial effect on human health [7,8]. The market dynamics, previously described, together with the technological development of avocado cultivation in tropical countries, has led to the continued growth of its planted area and production, with Colombia being one of the countries where this trend has been most marked [5]. The growth in the area planted with cv. Hass in tropical conditions has generated a need for knowledge about the physiological and phenological performance under these conditions. The knowledge will allow farmers to take advantage of its productive potential, seeking to overcome the barrier of 30 t ha^−1^ as the standard yield reported [3,9]. Despite the boom in cv. Hass, it is still considered that the current varieties behave as a species that has not been domesticated to commercial orchard environments, given the lack of knowledge of multiple aspects that govern its physiological behavior, which is highly influenced by the weather and by the relationships of the canopies with the rootstocks [3,9].

The avocado cv. Hass in tropical zones, in especial in countries as Colombia has gained importance as a relatively new crop, prompting research into its productive, phenological, and physiological behaviors [10]. The high edaphic and climatic variability under Colombia avocado production zones provides a range of microclimates, influencing growth patterns and fruit production indicating the necessitated an in-depth understanding of the crop's responses [11,12]. Phenological and physiological investigations have focused on the crop's responses to climatic stressors, such as water availability and temperature fluctuations [11,13,14]. These studies have facilitated the development of strategies to mitigate potential yield losses due to adverse environmental conditions.

It has been found that the production of avocado crop, as well as its quality, growth, development rates, phenological changes and, especially, processes governing productive and vegetative differentiation, are strongly related to water supply and temperature [[15], [16], [17], [18], [19], [20]]. Although there is basic knowledge about the effect of air temperature and soil humidity on several productivity variables, many factors and interactions remain unknown.

Under tropical conditions there are highly heterogeneous climatic variables such as air temperature and its derived concept, the accumulation of thermal units, which is expressed through thermal time (TT). This bio-climatic index, whose unit of measurement is the degree-day, can be obtained based on the threshold temperatures of growth (base temperature-BT) or physiological ranges and the air temperature at the local scale [[21], [22], [23]]. The duration of phenological stages, growth rates, and multiple physiological processes in plants are related to TT [24]. At higher temperatures at air and soil level (within the range of adaptation of the species), metabolic processes are accelerated, whereby the plant changes stage faster and its metabolic rates increase, a trend widely observed in species such as wheat and maize [25,26], and tropical fruits [14,27]. In these crops, the TT concept has been used as a basis for modeling aspects related to dates of emergence, sprouting, tillering, duration of vegetative periods, fruit filling, and ripening [28,29]. The ability to know and model these variables is important for the proper management of agricultural production systems. Similarly, TT-based models have been developed to determine the optimal timing for the application of plant growth regulators [30].

The phenological performance of crops can be evaluated by using development rates, mainly related to the duration of their respective stages and their relationship with TT [31]. Identifying the phenology of a species is an aspect of production system management that allows the implementation of agronomic management strategies, in addition to standardizing observations made in different environments and/or under different experimental conditions [32]. Specifically in the case of cv. Hass, the temperature is fundamental to its development and growth cycles. However, the information available for this fruit in tropical conditions is scarce, despite being it being one of the crops with the most active growth in planted areas worldwide. Knowing the relationship between phenological changes in avocado cv. Hass and TT could be of great value to farmers, since it would allow better planning and adequate establishment of new productive areas of this cultivar, achieving better resource management and facilitating models for harvest forecasting and implementation of different management practices according to phenology. Thus, the present study aimed to identify the variation and degree of association between the phenological behavior of avocado cv. Hass with TT and precipitation under tropical conditions.

## 2 Materials and methods

### 2.1 Location

The phenological observations supporting this study were carried out between 2015 and 2017, in six commercial plots of avocado cv. Hass grafted onto Antillean genotypes with a planting age of 5–7 years, distributed in five municipalities of the Antioquia department, Colombia (El Retiro, El Peñol, Amagá, Jardín, and San Pedro de Los Milagros). These municipalities cover three geographical sub-regions of the department (East, Southwest, and North). The following criteria were considered for the selection of the farms: (i) altitudinal range (1,700–2,500 m elevation); (ii) Hass monoculture system; (iii) farms with ICA registration for export; (iv) crops older than 5 years; and (v) optimal agronomic management. The selected farms were located between 1,740 and 2,453 m of elevation. Two were in the east of the Antioquia department ("LA" in El Peñol and "EG" in El Retiro), two in the southwest ("IM" in Amagá and "BV" in Jardín) and two in the north ("CS" and "EB" in San Pedro de Los Milagros). As a complement, two standard-producing farms (that met all the characteristics described above) were selected, one in each altitudinal profile: <2,200 and >2,200 orchards RIO (Rionegro, Colombia) and JER (Jerico, Colombia (Table 1). These plots are in life zones bh-MB (low montane humid forest), bmh-PM (very humid premontane forest), bmh-MB (very humid low montane forest), and bh-PM (premontane humid forest) [33].

**Table 1:** Location and meteorological conditions of contrasting localities as a function of elevation (2,200< masl and 2,200> masnn).

Location	Group	Region	Elevation (m)	Latitude	Longitude	Air Temperature (°C)			RH^a^ (%)	GSR^b^ (Watt m^−2^)
						Max.	Med.	Min.		
IM*	<2,200	Suroeste	1,753	06°01′42″	−75°40′42″	32,7	21,6	6,9	69,2	417,7
LA*		Oriente	2,009	06°11′40″	−75°13′59″	30,4	18,7	8,6	78,0	480,8
BV*		Suroeste	2,027	05°35′53″	−75°48′19″	29,8	18,4	12,3	81,5	387,5
JER**		Suroeste	1,856	05°47’41’’	-75°47’06”	28.5	17.8	11.9	80	420.6
CS*	>2,200	Norte	2,396	06°29′43″	−75°31′13″	27,2	15,4	2,5	79,8	438,2
EB*		Norte	2,453	06°29′26″	−75°31′28″	24,2	15,3	3,7	77,3	421,9
EG*		Oriente	2,288	06⁰02′51″	−75⁰29′42″	27,4	16,0	2,7	82,6	436,3
RIO**+		Oriente	2,250	06° 9' 8''	-75° 22' 48''	27.1	16.5	3.5	83.4	440.1

a RH= Relative Humidity.

b GSR = Global Solar Radiation. Max: Maximum. Med: median. Min: Minimum. *Plots selected for the determination of variations in duration and stoppage of phenological states and the determination of thermal time. **Plots selected for the analysis of growth flows in relative intensity. Specific location at Antioquia department, Colombia: LA: El Peñol; EG: El Retiro; IM: Amagá; BV: Jardín; CS and EB: San Pedro de Los Milagros, JER: Jericó and RIO: Rionegro.

The eight selected farms were divided into two groups for further analysis, where the main grouping criterion was elevation: (i) plots established <2,200 m (1,753 to 2,027 m), and (ii) plots established >2,200 m (2,250 to 2,453 m) (Table 1). The selection of these ranges and farms was based on homogeneity in the number of locations for the analyses and modeling performed. Also, these altitudinal ranges cover the areas where commercial crops of this production system are found in Colombia [12]. The evaluations were carried out in each lot every 8 days for 3 years (2015, 2016 and 2017), in which it was possible to obtain a series of 136 temporary observations of the phenological states, growth flows and monitoring of climatic variables. Additionally, there were three principal and secondary harvest cycles.

### 2.2 Weather conditions and measure

Weather data were generated using sensors located at observation points near the crop fields (less than 400 m away). The measuring devices were installed on a mast at heights ranging from 1.7 to 2.0 m above the ground surface. The sensors, together with the electrical autonomy system and the data storage unit, make up the automated weather station. Specifically, a WatchDog 2000® series station (Spectrum, IL), located in the field, was used in accordance with the World Meteorological Organization (WMO) guidelines for the installation of standard weather stations. Air temperature was measured in °C, air humidity in %, global solar radiation in MJ m^−2^, wind speed in m s^−1^ and precipitation in mm.

### 2.3 Selection of experimental units, evaluation, and description of growth fluxes

Within the six plots, 30 trees were selected and marked in the orchards, grouped in six blocks. To address the maximum variability available in the field, the blocking criterion was the degree of the terrain slope. The trees were identified with a unique code and georeferenced using a GPS (Garmin Ltd, USA). In addition, a Cartesian diagram of the tree was made, both vertically and horizontally, identifying the cardinal points (north, south, east, and west) and the height at which the reading was made. Then, four branches, on for each cardinal point, of the middle third of each tree were marked. Each of these was labeled in order to evaluate the phenological behavior over time. Sampling was carried out at intervals of 15 calendar days until reaching two years of evaluation, during which time the appearance of each of the different phenological stages of the crop was recorded. In each experimental unit, the code to which a given phenological stage corresponds was identified for the apical part of each of the four marked branches. This characterization was done according to the phenological growth flow diagram of the species according to the BBCH scale desing to cv, Hass (Biologische Bundesanstalt, Bundessortenamt und Chemische Industrie) [32].

For each evaluation period, the predominant phenological stage and its duration were determined. The present study focuses on six phenological stages, taking as selection criteria those with the greatest variation and that are unknown for tropical conditions. These consist of the following (i) principal growth stage 0 and 1: vegetative bud development and primary leaf expansion; (ii) principal growth stage 2: formation of axillary sylleptic buds, understood as the stage from the first visible sylleptic bud to the appearance of nine or more visible sylleptic buds; (iii) principal growth stage 3: primary bud extension, which starts from the beginning of bud extension until 90% or more of the final bud length is reached; (iv) principal growth stage 5: reproductive development, stating from the appearance of dormant reproductive buds until the end of inflorescence extension; (v) principal growth stage 6: flowering, from the opening of the first flowers until 90% or more of flowers open; and (vi) principal growth stage 7: fruit development [32].

Additionally, in each orchard selected (RIO and JER) at elevation classification (< and >2,200 m) the relative growth dynamics of different phenological phases and plant structures were evaluated over a period of one month to assess rates of growth and development. For this, four trees per farm were selected, and four branches of 1 m length, located in the middle part, directed towards each of the four cardinal points and with at least 10 shoots each, were labeled. The phenological phases were divided at the macro level into vegetative growth at the foliar and root level, flowering, development, and abscission fruit. The phenological stages were grouped according to the previously described BBCH scale [32]. All phenological growth states were transformed to relative intensities (RI). This process consists of assigning 100% to the maximum value of all the structures recorded during the evaluation period; where the RI results from dividing the value registered in each reading, by the maximum value multiplied by 100.

### 2.4 Determination of base temperature (BT) and thermal time (TT) calculation

The determination of the base temperatures for the phenological phases and for the complete cycle was carried out using four methodologies. The objective was to determine variation among the used methods, highlighting as the main objective the calculation of the TB for tropical conditions. It is important to mention that normally, BT is a parameter arbitrarily assumed as 10 °C as is reported in previous analysis [13], and calculated originally under latitudes other than tropical [34]. This approach does not consider that TB is a function of multiple environmental variables, especially in tropical conditions where elevation presents a substantial influence on the average temperature of the fields [14,23].

Based on the above premise, the methods evaluated were: (i) The least standard deviation in growing degree days (GDD); (ii) The least standard deviation in days; (iii) Coefficient of variation (CV) in days; and (iv) regression coefficient [35]. Additionally, a variation of method iii, called minimum CV in GDD, fitted to a second-degree regression model, was also evaluated [23]. For the calculation of BT with each method, the theoretical principles reported in the determination of TT were followed using a total of 25 base temperatures (from 0 to 12 °C with 0.5 °C intervals) [35]. The BT obtained according to the first four methods was determined using mathematical expressions for its calculation that have been validated under field conditions [35], which were implemented in the free software R [36]. The mathematical formulas and feeder variables are reported in Table 2.

**Table 2:** Method for base Temperature of avocado phenological stage calculation and mathematical model.

Method	Math equation
The least standard deviation in GDD	$x=\frac{\sum_{i=1}^{n}T_{i}d_{i}\sum_{i=1}^{n}d_{i}-n\sum_{i=1}^{n}d_{i}^{2}T_{i}}{{\left(\sum_{i=1}^{n}d_{i}\right)}^{2}-n\sum_{i=1}^{n}d_{i}^{2}}$
The least standard deviation in days	$x=T-\frac{{\left(\sum_{i=1}^{n}t_{i}d_{i}\right)}^{2}-n\sum_{i=1}^{n}t_{i}^{2}d_{i}^{2}}{n\sum_{i=1}^{n}d_{i}^{2}t_{i}-n\sum_{i=1}^{n}t_{i}d_{i}\sum_{i=1}^{n}d_{i}}$
Coefficient of variation in GDD	$x=\frac{\sum_{i=1}^{n}T_{i}d_{i}^{2}\sum_{i=n}^{n}T_{i}d_{i}-\sum_{i=1}^{n}d_{i}\sum_{i=1}^{n}T_{i}^{2}d_{i}^{2}}{\sum_{i=1}^{n}d_{i}^{2}\sum_{i=1}^{n}T_{i}d_{i}-\sum_{i=1}^{n}d_{i}\sum_{i=1}^{n}T_{i}d_{i}^{2}}$
Regression coefficient	$x=\frac{\sum_{i=1}^{n}T_{i}\sum_{i=1}^{n}d_{i}T_{i}-n\sum_{i=1}^{n}d_{i}T_{i}^{2}}{\sum_{i=1}^{n}d_{i}\sum_{i=1}^{n}T_{i}-n\sum_{i=1}^{n}d_{i}T_{i}}$

Ti = Mean temperature during the phenological stage at the field i; di = Duration of the phenological stage in the field i; ti = Difference between mean temperature of every field and the mean temperature of the phenological stage in the field i; T = Mean temperature of all the fields; n = number of fields. GDD: growing degree days.

To estimate the BT using the variation of method iii [23], the duration in days of each phase was recorded, as well as the summary of the daily mean temperatures recorded in each orchard for each phenological phase. Then, starting from a temperature of 0 °C, which was increased in intervals of 0.1 °C until reaching 12 °C, the base temperature (BT) was estimated for each of the phases considered. With the values obtained from the BT, the GDD was determined from equation (1) for each plot and for each phenological stage. The BT for each phase studied corresponds to the temperature for which the minimum value of the CV is obtained using a second-degree regression model, or where the first derivative of the regression model is equal to zero. The solution and determination of the equation (CV = f (BT)), was performed using the Solver tool for Excel®, by means of which the value of BT is found, where the first derivative is equal to zero. The method used is a non-linear iterative procedure that makes an initial approximation, varying the parameters until the minimum value of the sum of the squares of the deviations presented between the CVs calculated by the statistical method and the CVs calculated with the regression equation is found [23].

To find which method best determines BT, the CV was evaluated as a parameter for calculating variation, which expresses the relative variability of a data set. All calculations were performed by constructing functions in the free R software.

On the other hand, TT was calculated using two methods, in order to elucidate the variation between each one and the possible effects at the level of biological interpretation. These methods were (i) base temperature and (ii) physiological thresholds (maximum and minimum). The first methodology consists of the sum of the differences between the mean daily temperature and the base temperature (equation (1)). This method, better known as canonical, assumes that the growth and development of plants, and in general of ectotherms, can only be affected by low temperatures [37]. Related to the above, the use of this method also assumes that it is not necessary to know the intra-daily behavior of air temperature to determine reductions in development rates [37].

(1) $$GDD=\sum_{i=1}^{n}\left(Ti-BT\right)=\sum_{i=1}^{n}Ti-n*BT,\text{Where}$$

GDD: growing degree days; Ti: mean Temperature for the i day, BT: base temperature; n: phase duration in days.

The development of equations 2 and 3 consisted of the daily estimation of the thermal time by using threshold temperatures (minimum and maximum of 10 and 33 °C, respectively), reported for avocado cultivation [34], and the subsequent summation of these, according to the length of the cycle or phenological stage for which it has been determined. With this model, it is assumed that the growth and development rate of any ectotherm stops or ceases when the air temperature is below the minimum threshold or above the maximum threshold defined for the species [38], which agrees with Shelford's tolerance principle [39]. Due to the above, to make this calculation it is necessary to know the maximum and minimum temperature on a daily scale. With these temperatures, the daily behavior of air temperature is reconstructed, for which the simple triangle approximation was used [40], while the thermal time was generated using the vertical cut-off method, in which a total interruption of development is assumed at temperatures above or below the maximum and minimum threshold [38,41].

(2) $$GDD=\sum_{i=1}^{n}\left(\left(Tx+Tn\right)/2-BT\right),to\;Tx\;<\;Tc\;y\;Tn\;>\;BT$$

(3) $$GDD=\sum_{i=1}^{n}\left(\left(Tc+BT\right)/2-Tb\right),to\;Tx\;>\;Tc\;y\;Tn\;<\;BT),\text{where}$$

GDD: growing degree days, Tx: daily maximum air temperature, Tn: daily minimum air temperature, TB: low threshold-temperature (10 °C), Tc: high threshold-temperature (33 °C). n: phase duration in days.

### 2.5 Estimation of the rate of development and its relationship with temperature variations

In this case, specific BT values were determined for six phenological stages based on BBCH scales [32]. The development rate of the crop during each phenological stage was estimated and expressed as a function of time (days). The rate of development is equivalent to the frequency of occurrence of each phenological stage; that is, it is expressed as the reciprocal of time [31]. Frequency is a parameter used in different branches of science and engineering to specify the rate of occurrence of phenomena. That is, if a phenological stage in a locality requires, for example, 10 days, the rate of development will be equal to the frequency of occurrence of the state that characterizes this stage, i.e. 1/10 days or 0.1 day^−1^. The development rates were correlated with the mean minimum, mean, and mean maximum air temperatures of the analysis period for all sites [31]. Based on the relationships obtained, the effect of air temperature on the development rate of each of the phenological stages considered in the study was determined.

## 3 Results

### 3.1 Seasonality and duration of phenological stages for avocado cv. Hass has grown in tropical climates

**Fig. 1:**
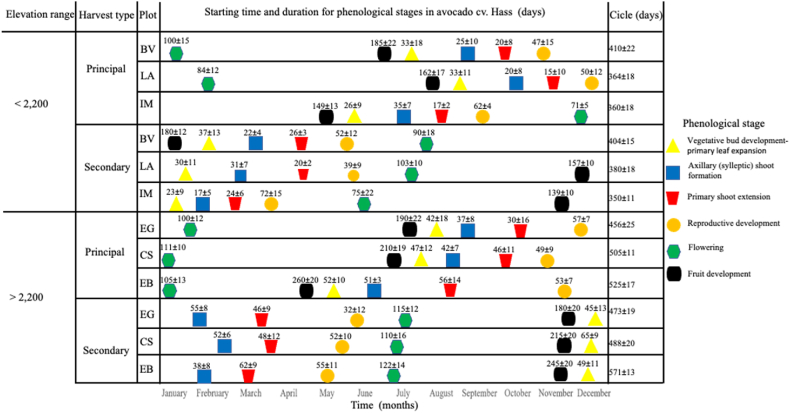
Dates of apparition and duration of phenological stages for avocado cv. Hass grown under six locations under tropical regions. Each symbol represents the predominant phenological state at the plot level based on the temporal dynamics of the BBCH scale for avocado without including the substages in each phase or the variation at the plant level. The phenological stages were based on BBCH classification for avocado cv. Hass [32], where phase 1 (Principal growth stage 0: vegetative bud development) and 1 (Principal growth stage 1: primary leaf expansion) were unified with the yellow symbol.

Fig. 1 represented the phenological dynamics at a monthly temporal scale where predominant phenological states at the plot level started, along with their temporal transitions to subsequent stages. Fig. 1 excludes the sub-states inherent to each stage. Moreover, it is important enounced that, on numerous occasions within the evaluation period, certain assessed branches did not complete all phenological stages. Alternatively, these branches could exhibit abrupt transitions, deviating from the sequential progression outlined by the BBCH scale.

In each cycle (principal and secondary harvest) and depending on its duration for each of the organized classes (>2,200 m<), an overlap was found between and within the phenological stages (Fig. 1). This situation generates that under tropical conditions in a plot there may be a predominant phenological state and sub-state, but at the same time it overlaps with another state (for example, fruit development (Stage 7)) and flowering (Stage 6)) and variations in sub-states (for example, No ovary growth visible and 90% or more of final fruit size).

When evaluating the stage of development in each of the plot, it was found that the phenological stages vary in duration within the orchards. Fig. 1 shows that there is a high variation in the occurrence of phenological stages independently of their locations and elevation range. However, it was determined that the duration of each phenological stage, between the vegetative and reproductive periods, varies with respect to elevation, both in the primary and secondary harvest type. Although there is a predominant stage for each time of year, the phenological stages at different altitudes overlap, which is expressed in a high interspecific variability at the level of lots, elevation range, and even individuals in each lot. On the other hand, in the elevation range <2,200 m, it is observed that the flowering stages are earlier than those of crops established above >2,200 m.

Regarding the productive cycles, it is evident that there are differences between crops planted above 2,200 m and those planted below 2,200 m, being much shorter in areas with higher temperatures. It was found that this tendency is conserved for both main and cross crops (Fig. 1). In addition, avocado cv. Hass crops within tropical environments at elevations exceeding 2,200 m results in reduced variability in both the duration and specific monthly timing of phenological phases, when contrasted with plots situated below the 2,200 m threshold (Fig. 1).

**Fig. 2:**
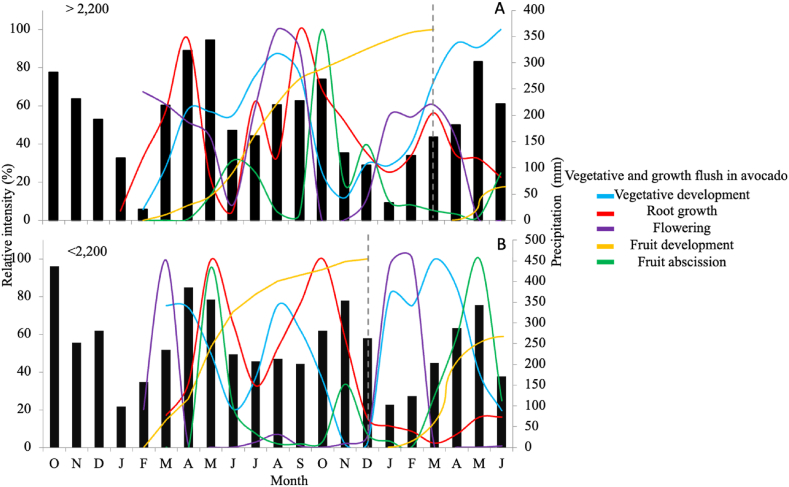
Dynamics of growth flows (vegetative buds, roots, flowers, and fruits) and fruits abscission in avocado cv. Hass under plots planted in tropical condition based in a representative of altitudinal profiles and precipitation influence. Specific location at Antioquia department, Colombia: A: Rionegro (>2,200); B: Jericó (<2,200). The vertical dotted gray line represents the division of the principal (left) and secondary (right) harvest.

**Fig. 3:**
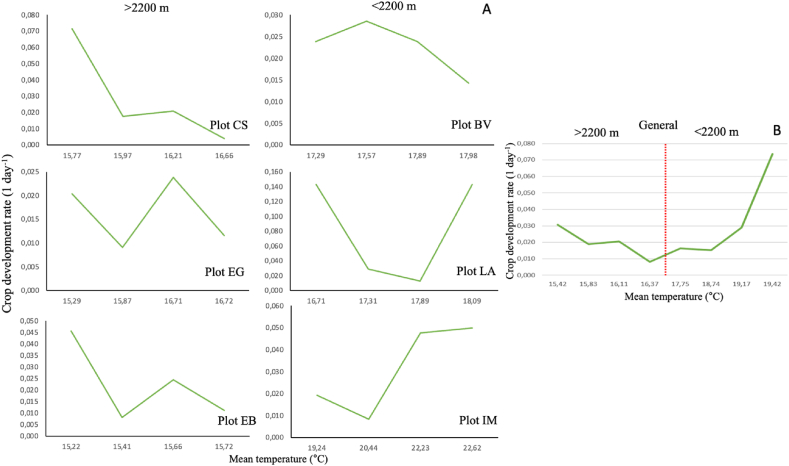
Behavior of crop development rate of avocado cv, Hass during a cycle as a function of temperature under tropical conditions. >2,200 m: plot at an altitude higher than 2,200 masl. <2,200: plot at an altitude lower than 2,200 masl. A: development rate for each locality. B: general development rate for all locations.

According to Fig. 1, for the principal harvest, the period between axillary bud formation and primary bud extension on farms below 2,200 m took approximately 30 days, while on farms above this altitude the time was approximately 60 days, indicating that vegetative development is accelerated at lower altitudes due to the influence of temperature (Table 1 and Fig. 2, Fig. 3). This situation is more marked if we analyze the period between the extension of the primary bud and reproductive development, which in the farms below 2,200 m was approximately 30 days, while in the others it was up to 120 days. The same trend was observed in the phenological behavior of the trees for the secondary harvests, corroborating the above. This influence on behavior of the elevation profile, associated with variables related to temperature (minimum, medium, and maximum), was more evident at the level of development of the fruit, where variations of almost 100 days were presented between the lowest (1,753 m) and highest (2,453 m) farms (Fig. 1).

The relative growth rates associated with phenological stages of the vegetative, flowering, and fruit fluxes, in addition to the dynamics of fruit uptake and root growth, corroborate the findings described above (Fig. 2 A and B). Additionally, we can identify that, depending on the elevation there are variations in the IR, its duration, and its occurrence according to the specific month of the year. In this regard, it can be identified that the precipitation may be a starter of some phenological processes, and there seems to be a close relationship between precipitation deficits and the induction of vegetative growth of leaves and roots, and flowering. Likewise, the highest growth rates of the fruit occur with increases in precipitation, and the abscission seems to coincide with the highest levels of rainfall (Fig. 1).

Furthermore, other identified patterns were linked to the observation that vegetative fluxes exhibit an upward trajectory after episodes of water stress induced by scant precipitation and elevated temperatures (drought). Notably, this trend appears to coincide with a discernible lag period of approximately 30–60 days with the peak incidence of fruit drop (abscission), suggesting a potential inverse relation between these developmental stages (Fig. 2 A and B). Similarly, RI of the fruit abscission variable aligns with the highest precipitation values, corresponding to the exponential growth phase of the fruit and coinciding in part (lag of 30 days approximately) with the peak levels of root development (Fig. 2 A and B).

### 3.2 Base temperature (BT) calculation for avocado cv, Hass under tropical conditions

The BT values obtained at each stage by each of the methods used were similar (Table 3). This means that, regardless of the mathematical expressions used, if there is an adequate selection of data and batches grouped by altitudinal profile in the case of tropical conditions, any of the methods used would generate reliable results. However, when comparing the coefficients of variation obtained for each method, it was found that the lowest variation was observed with the method of the coefficient of variation in days, with values of 7.9–9.8%, while the method with the greatest variation was the regression coefficient, which had values between 13.9 and 15.2% (Table 3).

**Table 3:** Base temperature associated with phenological stage for avocado cv. Hass grown under tropical regions according to the multiplex proposed method variation.

Method	Phenological stage	Base temperature (°C)	Variation coefficient (%)
The least standard deviation in GDD^1^	0–1	6.2	12.5
	1. 2	5.6	10.5
	3	0.9	13.5
	5	2.9	11.5
	6	1.9	10.4
	7	7.3	9.8
	CC*	6.4	11.1
The least standard deviation in days^1^.	0–1	6.4	15.7
	2. 2	6.1	13.4
	3	1.1	14.2
	5	3.1	12.4
	6	2.0	13.8
	7	7.5	12.8
	CC	6.6	12.1
Coefficient of variation in GDD^1^	0–1	6.1	7.8
	3. 2	5.45	9.8
	3	0.59	9,1
	5	2.8	8.8
	6	1.6	7.9
	7	7.3	6.9
	CC	6.3	8.2
Regression coefficient^1^	0–1	6.2	13.4
	2	5.2	14.8
	3	0.3	15.2
	5	2.5	13.9
	6	1.2	15.2
	7	7.0	16.7
	CC	6.3	14.5
Variation of Coefficient of variation in GDD^2^	0–1	6.1	
	4. 2	5.3	N.A
	3	0.3	N.A
	5	2.6	N.A
	6	1.3	N.A
	7	6.9	N.A
	CC	6.2	NA

Principal growth stage 0 and 2: vegetative bud development and primary leaf expansion; (ii) Principal growth stage 2: formation of axillary sylleptic buds, understood as the stage from the first visible sylleptic bud to the appearance of nine or more visible sylleptic buds; (iii) Principal growth stage 3: primary bud extension, which starts from the beginning of bud extension, until 90% or more of the final bud length; (iv) Principal growth stage 5: reproductive development, goes from the appearance of dormant reproductive buds, until the end of inflorescence extension; (v) Principal growth stage 6: flowering, from the opening of the first flowers until 90% or more of flowers open and (vi) Principal growth stage 7: fruit development [32]. ^1^Base temperature calculation method according to the equations reported in Table 2. Base temperature calculation method according to the proposed method variation^1^ [23].

In Table 3 there is evidence that base temperature values are specific for each phenological stage, indicating that each stage has certain requirements related to the temperature of the air surrounding the plant. According to the BT of each phenological stage described in Table 3, using the method of coefficient of variation in days, the descending order for each stage would be as follows: 7 (7.3 °C), 0–1 (6.1 °C), 2 (5.45 °C) > 5 (2.8 °C) > 6 (1.6 °C) > 3 (0.59 °C). The last of these needs only 10% of the full cycle temperature (0.59 °C), in contrast to 7 which requires almost the same temperature as that of the full cycle. It can also be observed that the phenological stages in which the highest air temperature is needed to complete each stage are 7 and 0–1. This is probably associated with the breaking of the dormant bud rest and the need for higher temperatures for the complete cycle of all phenological stages of the plant and minimum heat needs to achieve a minimum rate of growth and accumulation of bioactive components of the fruit. Meanwhile, the phenological stages that required the least air temperature for their development were 3, 5, and 6, which is possibly associated with a determinant or environmental factor for the starter or induction of these phases (Table 3).

### 3.3 Growing degree days (GDD)

In general, it was observed that, given the difference in BT for each phenological stage, the accumulation of thermal time implied a greater or lesser number of days in calendar time. This indicates that the thermal requirements for each stage are different. Regarding the GDD during the whole cycle, it was found that this value presented an important difference as a function of temperature. Specifically, for the <2,200 m group of plots, the GDD value varied between 1,621.7 and 3,236.6 (by the MTB method) (Table 4), with an average temperature of 19.57 °C (Table 1); while for the group >2,200 m this value was between 2,762.5 and 4,250.3 GDD (Table 4), with an average temperature of 15.5 °C (Table 1). These results also relate to the complete cycle length for both temperatures. On average, the duration of the cycle for the plots planted below 2,200 m was 9 months, while for the plots at higher altitudes, the duration was approximately 10 months. However, it should be noted that the greatest difference between the two environments is in the stage of extension of the primary shoot, since in the farms at higher altitudes this phase takes almost twice as long as in the lower altitudes.

**Table 4:** Growing degree day associated with phenological stage for avocado cv. Hass grown under tropical regions calculated by the physiological threshold (FT) and base temperature methods.

Phenological stage	Plots						Plots					
	<2,200 m						>2,200 m					
	IM		LA		BV		EG		CS		EB	
	FT	BT	FT	BT	FT	BT	FT	BT	FT	BT	FT	BT
0–1	185.4	425.2	600.1	1,450.7	170.2	376.4	269.1	701.4	215.6	602.5	88.4	198.7
2	169.25	350.4	511.96	1300.5	166.42	461.9	221.59	687.1	190.1	528.3	29,16	142.6
3	155.4	428.1	170.76	648.2	40.09	133.1	1,004.9	4,091.7	392.7	1,525.2	311,5	1845.9
5	828.85	1044,3	69.52	198.0	429.82	230.6	184.9	714.4	181.6	713.6	250,24	837.1
6	338.8	2,195.7	187.72	707.1	33.67	132.7	49.24	218.8	177.04	1,661.9	45,91	1,262.8
7	1200.1	2504.4	890.2	1900.1	680.9	1797.2	1,600.9	2,702.3	1,450.4	3,023.1	1,635.8	3207.7
CC	2877.8	3661.8	2,430.2	6,680.7	1,512.1	3,796.3	3,330.7	7,653.6	2,607.5	6,926.1	2,468.9	6,168.9

Principal growth stage 0 and 2: vegetative bud development and primary leaf expansion; (ii) Principal growth stage 2: formation of axillary sylleptic buds, understood as the stage from the first visible sylleptic bud to the appearance of nine or more visible sylleptic buds; (iii) Principal growth stage 3: primary bud extension, which starts from the beginning of bud extension, until 90% or more of the final bud length; (iv) Principal growth stage 5: reproductive development, goes from the appearance of dormant reproductive buds, until the end of inflorescence extension; (v) Principal growth stage 6: flowering, from the opening of the first flowers until 90% or more of flowers open and (vi) Principal growth stage 7: fruit development [32]. CC: complete cycle. FT: low threshold-temperature (10 °C) and high threshold-temperature (33 °C) [34]. BT: The values for the base temperature were those determined for each phenological state reported in Table 3 using the Coefficient of variation in GDD method. Specific location at Antioquia department, Colombia: LA: El Peñol; EG: El Retiro; IM: Amagá; BV: Jardín; CS and EB: San Pedro de Los Milagros.

Finally, when contrasting the GDD values obtained previously with those obtained by the triangulation method, it was found that these values were completely different. This is because this methodology uses a single BT for all phenological stages and is much higher than those obtained previously (14 °C).

### 3.4 Development rates

Under the analysis performed, the behavior of the development rate during the whole cycle for <2,200 m and >2,200 m (Fig. 3 A) depends on altitude. This variable remains relatively stable for >2,200, which has a lower environmental temperature, while for <2,200, this value tends to be higher and increase exponentially. In this sense, the behavior of the development rate of the different phenological stages of the crop in the >2,200 m plots had a similar trend, unlike those observed in the <2,200 m plots, which showed a slightly more heterogeneous behavior (Fig. 3 A). However, when the complete cycle is analyzed, with the average of the three locations for each altitude, it is observed that this development rate is more accelerated in lower environments than those found in the higher ones (Fig. 3 B).

## 4 Discussion

A high variation of phenological stages for avocado cv. Hass was found, indicating that both the appearance and duration of these stages are highly sensitive to environmental variables, such as temperature, humidity, precipitation, and sunshine, which were highly variable under the conditions of the evaluated plots (Table 1). It should be noted that temperature, being highly influenced by the altitudinal profile, showed high variation, thus modulating the duration of phenological stages at the lot and regional level under tropical conditions. In addition, a wide variety of phenological responses associated with the dates of emergence were found at the region, plot, and plant level, governed by possible climatic as precipitation and others triggers not considered in this study.

The present study demonstrates notable phenological disparities in avocado cv. Hass across distinct elevational strata. Specifically, variations in phenological stages were consistently more pronounced in areas plated below 2,200 m in comparison to those regions situated at altitudes exceeding 2,200 m. In the context of Colombia's tropical conditions, it appears that the upper cultivation areas (>2,200 m), predominantly characterized as highlands with relatively consistent topography, exhibit diminished climatic fluctuation [11]. Conversely, areas below 2,200 m present heightened topographical diversity and thus, an inclination towards microclimatic shifts [11], attributed in part to alluvial fans engendering variable wind patterns. Such winds, emanating from both warmer and colder zones, are contingent upon wind direction and flow dynamics.

Temperature, precipitation and other environmental variables play a crucial role in regulating the expression of genes and hormones synthesis involved in multiple physiological processes in avocados [42]. Specifically, low temperatures induce flowering in cv. Hass, while high temperatures promote floral organogenesis [42]. This phenomenon allows more spaced harvests and the possibility of producing fruit at different times, generating a competitive advantage in market terms under equatorial growing conditions, where avocado is grown in a wide altitudinal profile (1400–2700 m) [12]. As disadvantages it can be stated that this wide altitudinal profile can generate variations in quality parameters, and adaptation of the species to different climatic conditions [11,14].

In tropical under rainfed conditions, the vegetative growth dynamics of avocados cv. Hass, encompassing both root, leaf, and fruit development, is intricately interlinked with the precipitation [43]. The availability of water in the soil assumes a central role in shaping the competition for resources between vegetative growth processes and fruit sets impacting avocado productivity [44]. This allocation of resources, in turn, affects the overall health and vitality of the avocado tree and directly influences the development of fruits. Therefore, understanding the intricate balance between water availability, vegetative growth, and fruit set is crucial to design effective cultivation strategies and improving yield under tropical avocado production. One prospective remedy for this technological lag could involve the implementation of irrigation systems, however, a dearth of precise and reproducible data persists regarding the impact of irrigation on yield constituents and the phenological patterns of avocado cv. Hass across diverse production zones in Colombia. Initial investigations and climate-based simulations pertaining to the water equilibrium of this avocado cultivar have yielded insights [44]. Notably, it was deduced that a substantial portion of the producing regions experience a minimum of one month characterized by water insufficiency, thereby necessitating timely irrigation interventions [44].

Regarding the determination of the base temperature, the values were quite different from those reported in the literature and generalized by decision-makers, who use a value of 10 °C, independently of the area and the specific climatic conditions in the equatorial region [13]. Meanwhile, the values obtained in our work ranged between 0.3 and 7.5 °C, depending on the different phenological stages evaluated, indicating that cv. Hass in tropical conditions does not develop below 0.3 °C. This implies that, from a practical perspective, planting at altitudes above 2,500 m in areas with latitude above three degrees and below 2,700 in areas between 0 and 3° latitude should be discouraged [45]. This is because, under these conditions, air temperatures below this value (0.3 °C) can be found. This information is essential for the programming and execution of different cultural tasks and for the proper management of the crop, as well as in the future generation of a predictive model of harvests based on specific agroclimatic conditions.

The different phenological phases presented variations in their base temperatures. This could be associated with the fact that temperature is one of the main factors in the change from the vegetative to the reproductive phase. Subtropical avocado cultivars such as cv. Hass are considered to produce flower buds only if they are maintained under a cold temperature regime [[46], [47], [48]]. Furthermore, our results agree with previous work, where it is reported that in cv. Hass the onset of flowering is preceded by low temperatures and, even in arid conditions, low temperatures are a key factor in the transition of buds to the reproductive phase [49]. Although precipitation plays a key role, in subtropical conditions it has been found that low temperatures as an inducer of flowering cannot be replaced by periods of water deficit [49], but under our findings, these two variables are related, where flowering seems to be associated with low temperatures and water deficit.

In the context of avocado responses to temperature fluctuations, exposure to elevated temperatures has been observed to trigger a reduction in the photosynthesis rate, the accumulation of photosynthetic pigments, and a decrease in water use efficiency in plants [50]. Additionally, throughout the budding and flowering phases, variations in temperature have the capacity to impact the synthesis and accumulation of phytohormones, specifically auxins and gibberellins, which play essential roles in the growth and differentiation of flower buds [42]. Furthermore, extreme temperature conditions, whether high or low, can induce alterations in the opening pattern of male and female flowers, ultimately affecting the flowering period's duration and the total flower count [48].

Conversely, during the stages of fruit set and development, temperature emerges as a pivotal factor influencing the biosynthesis of fatty acids within avocado [51]. Elevated temperatures tend to elevate enzyme activity, potentially leading to heightened lipid production. However, excessively high temperatures can result in enzyme denaturation and consequent loss of activity, subsequently causing a decline in lipid synthesis [51].

According to the results, it can be deduced that cv. Hass under high tropic conditions maintains the characteristics of its genetic origin (mostly Guatemalan) in terms of its tolerance to cold, which is in agreement with reports that mention that this cultivar can withstand temperatures as low as −2 °C without damage to different parts of the plant [3,9,52]. It should be noted that one of the main limitations in avocado cultivation is the exposure of the tree to low temperatures, since, being a persistent-leaf fruit tree, this can cause damage to the foliage, flowers, and even fruit [3,53].

Based on the results, it is inferred that there is a particular climatic zone in Colombia that, due mainly to its geographical location in latitudes very close to the equator, contrasts with the conditions reported in the literature worldwide for the cultivation of avocado cv. Hass, such as cool semi-arid climates with predominant rainfall in winters, humid subtropical climates with rainfall predominantly in summer, and tropical and subtropical climates with predominant rainfall in summer [3]. This zone, predominant in avocado cv. Hass production zones in Colombia, could be called a high Andean tropical climate with two marked rainy periods alternating with dry periods and with average temperatures with variation associated with the altitudinal profile. In addition, our results agree with those that found that the phenological development of avocado is influenced by precipitation, air humidity, altitude, and other factors in addition to air temperature [43], with specific variations at the farm level in terms of duration, appearance and overlap between phenological phases.

The physiological periods in the shoot formation stage at post-anthesis are in the range of variation found in other latitudes. Specifically, the <2,200 m group of plots (areas of higher temperature) presents a duration of 174.7 d, similar to a semi-arid Mediterranean climate such as that of California, which had a duration of 225 d, while the >2,200 m group (lower temperature zones) had a duration of 307.3 d, behavior similar to a semi-hot sub-humid climate such as that of Nayarit with a duration of 360 d (Table 4) [54]. Temperature usually defines the range within which avocado is commercially grown under tropical conditions. In this regard, based on our result it has been established that the period between flowering and harvest varies according to the altitude and temperature of the crop. In this sense, in localities with mean temperatures of 20, 19, 17, and 14.7 °C, the time from flowering to harvest was 8–9, 10–11, 11–12, and 12–13 months respectively (Fig. 3). Likewise, the duration of the growth and development cycles of the fruit varied in a range between 140 and 250 days, depending of TT, coinciding with the duration of another areas worldwide, from the coldest (New Zealand), to the warmest, such as Mediterranean climates (California, Chile, Spain, among others) [3].

The base temperature found in the present study, according to the different models for the entire cycle, fluctuated between 5.4 and 6.3 °C, with an average of 5.86 °C. This result contrasts with that reported by several authors, who mention that the base temperature for Hass avocado is 10 °C [13]. This is possibly due to the fact that, under tropical conditions in high Andean zones where Hass avocado is produced, periods of temperature below 10 °C are infrequent and last for very short periods, allowing the plant to grow below this threshold without affecting its development and productivity. In the areas under study, there were not many events with temperatures above 32 °C, reported as the upper range [34]. Such temperatures have negative effects due to their detrimental impact on fertilization, the degree of pollen sterility, and other factors.

Elevation (meters above sea level) was an important variable when selecting sampling sites to our analysis. Altitudinal contrasts ensured significant differences in the environmental conditions where avocado orchards grow, and specifically in air temperature [11]. However, in addition to significant changes in temperature, other environmental changes related to altitudinal variation can affect plant growth and development. In this context, a potential scenario might involve an elevation-dependent increase in UV radiation values, establishing an inverse correlation with temperature variations across the altitudinal gradient [55]. For example, UV radiation, capable of affecting physiological mechanisms in cultivated species, has a strong and negative relationship with altitude [14,56]. However, with a difference of 700 m between the highest and lowest sites and the high cloud cover of the study region, the differences in UV radiation between the highest and lowest sites are negligible (Table 1).

Despite the substantial expansion of avocado cultivation in tropical regions [5], marked variability persists in its physiological, phenological, and productive characteristics as was evidenced in our study. This stands in stark contrast to regions like intertropical, Mediterranean, and desert zones, where more uniform climate and soil conditions prevail, resulting in a more standardized behavior of the avocado species [3]. The heightened diversity of responses observed in tropical zones underscores the intricate interplay between the species and the multifaceted tropical environment, encompassing intricate climatic gradients and heterogeneous soil compositions. This phenomenon highlights the need for nuanced cultivation strategies tailored to the dynamic and heterogeneous tropical context, diverging from the more homogenized conditions of other growth areas worldwide.

## 5 Conclusions

The duration of the phenological stages of avocado cv. Hass under tropical conditions varies according to altitude, which leads a high inter-specific variability in regions, lots, and even individual trees. Production cycles are shorter in areas with higher temperatures and longer in areas with lower temperatures. There is no single minimum threshold temperature for avocado cv. Hass in the Colombian high tropics, since it is specific for each phenological stage. In addition, this cultivar does not develop below 0.3 °C, which has been found to be the minimum base temperature, which implies that its plantation should not be established at altitudes above 2,500 m above sea level, where these temperatures usually occur. On the other hand, the development rate of avocado cv. Hass is more accelerated at higher temperatures, and at lower temperatures, there are longer sprouting periods.

## Funding statement

No funding was received for this work.

## Author contribution statement

Conceived and designed the experiments and Performed the experiments: Joaquín Guillermo Ramirez-Gil, Cipriano Arturo Diaz-Diez and Jorge Alonso Bernal-Estrada.

Analyzed and interpreted the data: Joaquín Guillermo Ramírez-Gil, Juan Camilo Henao Rojas, Cipriano Arturo Diaz-Diez, Andrés Javier Peña-Quiñones, Nicolas León, Alfonso Parra-Coronado, Jorge Alonso Bernal-Estrada.

Contributed reagents, materials, analysis tools or data and Wrote the paper: Joaquín Guillermo Ramírez-Gil, Juan Camilo Henao-Rojas, Cipriano Arturo Diaz-Diez, Andrés Javier Peña-Quiñones, Nicolas León, Alfonso Parra- Coronado, Jorge Alonso Bernal-Estrada.

## Data availability statement

Data will be made available on request.

## Declaration of interest's statement

The authors declare no conflict of interest.

## Additional information

No additional information is available for this paper.

## Declaration of competing interest

The authors declare that they have no known competing financial interests or personal relationships that could have appeared to influence the work reported in this paper.
